# Supplementary material for: Rab11 endosomes and Pericentrin coordinate centrosome movement during pre-abscission in vivo
Source: Life Sci Alliance. 2022 Mar 18;5(7):e202201362. doi: 10.26508/lsa.202201362 (PMC8933627; doi:10.26508/lsa.202201362)
Supplement: Supplementary file 3 [file LSA-2022-01362_TableS1.docx]

| **Figures** | **Y-Axis** | **Category** | **n**  **(cell)** | **n**  **(embryo)** | **n**  **(experiment)** | **Statistical Test** | **Parameters** | **Result** | **p-value** |
| --- | --- | --- | --- | --- | --- | --- | --- | --- | --- |
| 1E | Number of centrosomes with bridge directed centrosome movement in pre-abscising cells | Zebrafish embryo cell (Centrin-GFP) | n=26 | n=4 | N/A | One Way ANOVA | F (4,102) = 0.82 | ** | 0.0082 |
|  |  | Zebrafish embryo cell (PLK1-mCh) | n=40 | n=4 | N/A |  |  |  |  |
|  |  | Zebrafish embryo cell in the Kupffer’s Vesicle | n=10 | n=2 | N/A |  |  |  |  |
|  |  | Human cell (HeLa) Centrin-GFP | n=15 | N/A | n=3 |  |  |  |  |
|  |  | Human cell (HeLa) Ds-Red-PACT | n=21 |  | n=4 |  |  |  |  |
| 1E | Number of centrosomes per pre-abscising cell with bridge directed centrosome movement | Zebrafish embryo cell (Centrin-GFP) | n=26 | n=4 | N/A | One Way ANOVA | F (2,71) = 0.03110 |  |  |
|  |  | Zebrafish embryo cell (PLK1-mCh) | n=40 | n=4 | N/A |  |  | n.s. | 0.9973 |
|  |  | Zebrafish embryo cell in the Kupffer’s Vesicle | n=10 | n=2 | N/A |  |  | n.s. | 0.7506 |
|  |  | Human cell (HeLa) Centrin-GFP | n=15 | N/A | N/A | Two-tailed Student's t-test | t = 0.2392, df = 31 | n.s. | 0.8126 |
|  |  | Human cell (HeLa) Ds-Red-PACT | n=21 |  | N/A |  |  |  |  |
| 1E | Number of centrosomes per pre-abscising cell with bridge directed centrosome movement | Zebrafish embryo cell (Centrin-GFP) | n=26 | n=4 | N/A | Two-tailed Student's t-test | t = 3.365, df=80 | ** | 0.0012 |
|  |  | Zebrafish embryo cell (PLK1-mCh) | n=40 | n=4 | N/A |  |  |  |  |
|  |  | Zebrafish embryo cell in the Kupffer’s Vesicle | n=10 | n=2 | N/A |  |  |  |  |
|  |  | Human cell (HeLa) Centrin-GFP | n=15 | N/A | N/A |  |  |  |  |
|  |  | Human cell (HeLa) Ds-Red-PACT | n=21 |  | N/A |  |  |  |  |
| 2C | Fold change of photoconverted Dendra-Rab11 increase at the cytokinetic bridge | Before Photobleaching | n=6 | N/A | n=4 | Two-tailed Student's t-test | t = 6.029, dF = 10 | *** | 0.0001 |
|  |  | After Photobleaching |  |  |  |  |  |  |  |
| 3C | % Centrosome movement towards the cytokinetic bridge first | Human (HeLa) Cells | n=10 | N/A | N/A | N/A | N/A | N/A | N/A |
|  |  | Zebrafish embryo cell at epiboly | n=13 | n=4 | N/A |  |  |  |  |
| 3E | Fluorescent Intensity (Normalized) | Oldest centrosome | n=3 | N/A | N/A | N/A | N/A | N/A | N/A |
|  |  | Youngest Centrosome |  |  |  |  |  |  |  |
| 3F | Oldest centrosome/ Youngest centrosome | Mobile Fraction (%) | n=3 | N/A |  | One-Way ANOVA | F (2,6) = 7.515 | * | 0.016 |
|  |  | Half Life (T1/2) |  |  |  |  |  | n.s. | 0.49 |
| 3J | Endosome Area at Daughter or Mother Centriole | Oldest Centrosome | n=7  Centrosomes | | N/A | Two-tailed Student's t-test | t=4.268, df=12 | ** | 0.0011 |
|  |  | Youngest Centrosome |  |  |  |  |  |  |  |
| 4C | Number of centrosomes per pre-abscising cell with bridge directed centrosome movement | Control | n=25 | N/A | N/A | One Way ANOVA | F (4,79) = 3.148 |  |  |
|  |  | Rab11-null cells | n=35 |  | N/A |  |  | ** | 0.0049 |
|  |  | Rab11-null cells, plus mCh-Rab11 | n=7 |  | N/A |  |  | n.s. | 0.9991 |
|  |  | Rab11-null cells, plus mCh-Rab11(S25N) | n=9 |  | N/A |  |  | ** | 0.0073 |
|  |  | Rab11-null cells, plus mCh-Rab11(Q70L) | n=6 |  | N/A |  |  | *** | 0.0005 |
| 4F | Directional displacement of centrosome towards the cytokinetic bridge (μm) | Control | n=9 | N/A | N/A | One Way ANOVA | F (4, 87) = 6.21 |  |  |
|  |  | Rab11-null cells | n=15 |  | N/A |  |  | *** | 0.0002 |
|  |  | Rab11-null cells, plus mCh-Rab11 | n=7 |  | N/A |  |  | n.s. | 0.1019 |
|  |  | Rab11-null cells, plus mCh-Rab11(S25N) | n=9 |  | N/A |  |  | *** | 0.0009 |
|  |  | Rab11-null cells, plus mCh-Rab11(Q70L) | n=6 |  | N/A |  |  | *** | 0.0006 |
| S3F | % Binucleated cells | Control | n>100 | N/A | n=3 | One Way ANOVA | F (2,6) = 18.07 |  |  |
|  |  | Rab11-null cells | n>100 |  | n=3 |  |  | ** | 0.0021 |
|  |  | Rab11-null cells, plus mCh-Rab11 | n>100 |  | n=3 |  |  | n.s. | 0.3245 |
| S3G | Total distance traveled by centrosome post anaphase exit (μm) | Control | n=9 | N/A | n=4 | Two-tailed Student's t-test | t=5.000, df=32 | **** | <0.0001 |
|  |  | Rab11-null cells | n=8 |  | n=4 |  |  |  |  |
| S3I | Mean Fluorescent Intensity (A.U.) | Rab11-null cells, plus mCh-Rab11 | n=18 | N/A | N/A | Two-tailed Student's t-test | t=3.208, df=30 | n.s. | 0.2183 |
|  |  | Rab11-null cells, plus mCh-Rab11(Q70L) | n=15 |  |  |  |  |  |  |
| S3J | Fluorescent Intensity Normalized (A.U.) | Rab11-null cells, plus mCh-Rab11 | n=18 | N/A | N/A | N/A | N/A | N/A | N/A |
|  |  | Rab11-null cells, plus mCh-Rab11(Q70L) | n=15 |  |  |  |  |  |  |
| S3K | Mobile Fraction (%) | Rab11-null cells, plus mCh-Rab11 | n=18 | N/A | N/A | Two-tailed Student's t-test | t=3.208, df=30 | ** | 0.0032 |
|  |  | Rab11-null cells, plus mCh-Rab11(Q70L) | n=15 |  | N/A |  |  |  |  |
| S3L | Half-life (t1/2, sec) | Rab11-null cells, plus mCh-Rab11 | n=18 | N/A | N/A | Two-tailed Student's t-test | t=4.594, df=31 | **** | <0.0001 |
|  |  | Rab11-null cells, plus mCh-Rab11(Q70L) | n=14 |  | N/A |  |  |  |  |
| 5B | Ratio of Rab11-null centrosome intensity / Control intensity (A.U.) | Centrin | n>30 Centrosomes/  experiment | | n=3  n=3 | One Way ANOVA | F (4, 10) = 16.55 | n.s. | 0.9991 |
|  |  | Cenexin |  |  |  |  |  |  |  |
|  |  | Pericentrin |  |  | n=3 |  |  | * | 0.0352 |
|  |  | GFP-FIP3 |  |  | n=3 |  |  | *** | 0.0008 |
|  |  | Transferrin Receptor |  |  | n=3 |  |  | *** | 0.0004 |
| 5D | Pericentrin normalized intensity pre-abscising cells (A.U.) | Control | n=38 Centrosomes | | n=5 | One Way ANOVA | F (4, 242) = 18.19 |  |  |
|  |  | Rab11-null cells | n=43 Centrosomes | | n=5 |  |  | **** | <0.0001 |
|  |  | Rab11-null cells, plus mCh-Rab11 | n=62 Centrosomes | | n=4 |  |  | n.s. | 0.6632 |
|  |  | Rab11-null cells, plus mCh-Rab11(S25N) | n=61 Centrosomes | | n=4 |  |  | **** | <0.0001 |
|  |  | Rab11-null cells, plus mCh-Rab11(Q70L) | n=43 Centrosomes | | n=3 |  |  | **** | <0.0001 |
| S4G | Pericentrin normalized intensity in late pre-abscising cells (A.U.) | Control | n=22 Centrosomes/  experiment | | n=5; 1 represen-tative shown | Two-tailed Student's t-test | t=11.00, df=42 | **** | <0.0001 |
|  |  | Rab11-null Cells | n=20 Centrosomes/  experiment | |  |  |  |  |  |
| 6C | Number of centrosomes per pre-abscising with bridge directed centrosome movement | Control | n=26 | n=4 | N/A | One Way ANOVA | F (3,77) = 2.733 |  |  |
|  |  | PCNT+/- | n=16 | n=4 |  |  |  | ** | 0.0025 |
|  |  | Centrin-GFP, plus CRY2 | n=21 | n=4 |  |  |  | n.s. | 0.6449 |
|  |  | Centrin-GFP, plus CRY2 and CIB1-mCh-Rab11 | n=19 | n=4 |  |  |  | * | 0.0126 |
| 6D | Distance traveled by centrosome after anaphase (μm) | Control | n=19 | n=4 | N/A | One Way ANOVA | F (3,110) = 9.907 |  |  |
|  |  | PCNT+/- | n=14 | n=4 |  |  |  | *** | 0.0001 |
|  |  | Centrin-GFP, plus CRY2 | n=14 | n=4 |  |  |  | n.s. | 0.8335 |
|  |  | Centrin-GFP, plus CRY2 and CIB1-mCh-Rab11 | n=11 | n=4 |  |  |  | ** | 0.0067 |
| 6F | Daughter cells with supernumerary centrosomes | Control | n>30/embryo | n=3 | N/A | One Way ANOVA | F (3,8) = 38.51 |  |  |
|  |  | PCNT+/- | n>30/embryo | n=3 |  |  |  | *** | 0.0003 |
|  |  | Centrin-GFP, plus CRY2 | n>25/embryo | n=3 |  |  |  | n.s. | 0.5719 |
|  |  | Centrin-GFP, plus CRY2 and CIB1-mCh-Rab11 | n>35/embryo | n=3 |  |  |  | *** | 0.0003 |
| S5G | % Binucleated cells | Control | n>42/embryo | n=13 | N/A | One Way ANOVA | F (3,25) = 5.644 |  |  |
|  |  | PCNT+/- | n>42/embryo | n=9 |  |  |  | * | 0.0417 |
|  |  | plus CIB1-mCh-Rab11 | n>30/embryo | n=3 |  |  |  | n.s. | 0.9916 |
|  |  | plus CRY2 and CIB1-mCh-Rab11 | n>30/embryo | n=4 |  |  |  | ** | 0.0031 |

**Table S1. Detailed statistical analysis results reported.**

**SUPPLEMENTARY KEY RESOURCE TABLE**

| **Reagent or RESOURCE** | **Source** | **Identifier** |
| --- | --- | --- |
| **Antibodies** | | |
| Rab11 | Cell Signaling Technologies | 3539S; RRID: AB_2253210 |
| Gamma-tubulin | SigmaAldrich | T5192; RRID: AB_261690 |
| GAPDH-HRP | Proteintech | HRP-60004; RRID: AB_2737588 |
| Donkey Anti-Rabbit-HRP | Jackson Immuno Research | 711-035-152; RRID: AB_10015282 |
| Transferrin Receptor | Abcam | ab1086; RRID: 297535 |
| Pericentrin | Abcam | ab4448; RRID: AB_304461 |
| Cenexin | ProteinTech | 12058-1-AP; RRID: AB_2156630 |
| Anti-GFP | GeneTex | GTX13970; AB_371416 |
| ZO-1 Monoclonal Antibody (ZO-1, 1A12) Alexa Fluor 488 | Life Technologies | 339188; RRID: AB_2532187 |
| Alexa Fluor Anti-Rabbit 488 | Life Technologies | A21206; RRID: AB_2535792 |
| Alexa Fluor Anti-Rabbit 568 | Life Technologies | A10042; RRID: AB_2534017 |
| Alexa Fluor Anti-Rabbit 647 | Life Technologies | A31573; RRID: AB_2536183 |
| Alexa Fluor Anti-Mouse 488 | Life Technologies | A21202; RRID: AB_141607 |
| Alexa Fluor Anti-Mouse 568 | Life Technologies | A10037; RRID: AB_2534013 |
| Alexa Fluor Anti-Mouse 647 | Life Technologies | A31571; RRID: AB_162542 |
| Anti-aTubulin antibody Alexa Fluor 555conjugated | EMD millipore | 05-829-AF555 |
| **Chemicals, Peptides, and Recombinant Proteins** | | |
| DAPI | SigmaAldrich | D9542-10mg |
| NucBlue^TM^ Fixed Cell stained Ready Probes | ThermoFischer | R37606 |
| NucBlue^TM^ Live Ready Probes | ThermoFischer | R37605 |
| Agarose | ThermoFischer | 16520100 |
| BSA | Fisher Scientific | BP1600-100 |
| BIO BASIC Maxi Prep Kit | BIO BASIC | 9K-0060023 |
| Dimethylsulphoxide | Fisher Scientific | BP231-100 |
| Paraformaldehyde | Fisher Scientific | O4042-500 |
| Phosphate Buffered Saline | Fisher Scientific | 10010023 |
| Life Technologies Prolong Diamond Antifade Moutant with DAPI | Fisher Scientific | P36971 |
| 35 mm Dish\| No.1.5. coverslip\| 20 mm Glass Diameter | MatTek Corporation | P35G-1.5-20-C |
| μ-Slide 8 Well Glass Bottom: No. 1.5H (170 μm +/- 5 μm) D263 M Schott glass, sterilized | IBIDI | 80827 |
| Molecular Probes Prolong Gold Antifade Moutant | Fisher Scientific | P36934 |
| Triton X-100 | Fisher Scientific | BP151500 |
| Tween 20 | ThermoFischer | BP337500 |
| Tris-HCl | Fisher Scientific | BP153 |
| APS | Fisher Scientific | BP179-100 |
| 40% Acrylamide | Sigma-Aldrich | A4058-100ML |
| 97% Sodium acrylate | Sigma-Aldrich | 408220-25G |
| 40% Bisacrylamide | EMD Millipore | 1300-500ML |
| TEMED | Fisher Scientific | BP150-100 |
| Disposable Biopsy Punch | Integra Miltex | 33-34 |
| Ethylenediaminetetraacetic acid (EDTA) | Fisher Scientific | BP120 |
| Sodium Chloride | Fisher Scientific | BP358 |
| NEBuilder HiFi DNA assembly Cloning Kit | New England BioLabs | E5520S |
| mMESSAGE mMACHINE^TM^SP6 | Invitrogen | AM1340 |
| Mirus TransIT-LT1 transfection | Mirus | MIR2305 |
| Rab11A CRISPR Vector | Santa Cruz Biotechnology | SC-400617 |
| Rab11A HDR Vector | Santa Cruz Biotechnology | SC-400617-HD |
| BioRad Protein Assay Kit II | BioRad Laboratories | 5000002 |
| Ponceau Stain | Boston BioProducts | ST-180-500 |
| Clarity^TM^ Western ECL substrate | BioRad Laboratories | 170560 |
| **Experimental models: Cell lines** | | |
| HeLa Cells with stable GFP-FIP3 | Hehnly and Doxsey 2014; Hehnly et al., 2012; Wilson et al., 2005 | N/A |
| HeLa Cells with stable Centrin-GFP | Kuo et al., 2011; Piel et al., 2001 | N/A |
| Rab11KO HeLa Cells with stable GFP-FIP3 | Rathbun et al., 2020a | N/A |
| Rab11 KO HeLa Cells with stable Centrin-GFP | This paper | N/A |
| **Experimental models, organisms and strains** | | |
| Zebrafish | Zebrafish International Resource Center | TAB-Wildtype |
| Zebrafish | Zebrafish International Resource Center | Tg (Sox17:DsRed) |
| Zebrafish | Gift from Solinca-Krezel lab, generated by Harris Lab | Tg (-5actb2:cent4-GFP) |
| Zebrafish | Sepuldeva et al., 2018. Gift from Jao Lab UCSD | Tg (*pcnt*^tup2^) |
| Zebrafish | (Dasgupta and Amack, 2016) | Tg (sox17:GFP-CAAX)^sny101^ |
| Zebrafish | (Navis et al., 2013) | Tg*BAC*(cftr-GFP) |
| Zebrafish | This paper | Tg (-5actb2:cent4-GFP), Tg (*pcnt^tup+^*^/-^) |
| **Recombinant DNA** | | |
| Plasmid: PCS2-CRY2 | (Rathbun et al., 2020a | Addgene Plasmid #140572 |
| Plasmid: PCS2-CIB1-mCherry-Rab11a | Rathbun et al., 2020a) | Addgene Plasmid #140573 |
| Plasmid: PCS2-CIB1-mCerulean-Rab11a | (Rathbun et al., 2020a) | Addgene Plasmid #140574 |
| Plasmid: mRuby2-MannII-N-10 | (Lam et al., 2012) | Addgene Plasmid #55903 |
| Plasmid: PCS2-mCherry-Rab11a | This paper | Addgene in process |
| Plasmid: PCS2-mCherry-Rab11a (S25N) | This paper | Addgene in process |
| Plasmid: PCS2-mCherry-Rab11a (Q70L) | This paper | Addgene in process |
| Plasmid: PCS2-Dendra-Rab11a | This paper | Addgene in process |
| Plasmid: PCS2-PLK1-mCherry | (Colicino et al., 2019) | Addgene Plasmid #127154 |
| **Software and algorithms** | | |
| ImageJ/FIJI | NIH and Laboratory for Optical and Computational Instrumentation | https://imagej.net/Fiji |
| IMARIS, Bitplane | Oxford Instruments | https://imaris.oxinst.com/ |
| PRISM9 | GraphPad | https://www.graphpad.com/scientific-software/prism/ |
| LAS-X Software | Leica Microsystems | https://www.leica-microsystems.com/products/microscope-software/p/leica-las-x-ls/ |
| VisiView | Visitron | https://www.visitron.de/products/visiviewr-software.html |
| AutoQuant X3 | Meyer Instruments | https://www.meyerinst.com/mediacybernetics/autoquant/ |
| Zeiss Zen 3.2 | Carl Zeiss | https://www.zeiss.com/microscopy/us/products/microscope-software/zen.html |
